# The Chicago Medical Society

**Published:** 1870-07

**Authors:** 


### THE CHICAGO MEDICAL SOCIETY.

This Society holds its regular meetings during the summer, on the first and third Monday of every month.

The subject for discussion, on June 20th, was the constipation of infants, its causes and medical treatment. Drs. Quine, Paoli, Fitch, Davis, and Gray expressed their views. They all condemned the use of strong purgatives and injections; the former as liable to increase the difficulty, and the latter to distend and weaken the muscular fibres.

Dr. Paoli had used a wax bougie, to excite the peristaltic action of the bowel, and recommended suppositories of tallow or soap, for the same purpose. Dr. Fitch had found much benefit from electricity, applied to the back and along the course of the colon, also from a prescription containing—

℞. Tinct. Aloes,
Syrup Ipecac,
Fl. Ex. Rhubarb.

Dr. Davis believed much of the difficulty arose from a lack of the peristaltic action of the bowel, and to stimulate their fibres, often used with a beneficial effect minute doses of tinct. nux vomica, perhaps, 1 drop; syrup ipecac, perhaps, 5 drops; and might add, sometimes, with benefit, syrup rhubarb or tinct. belladonna.

Dr. Gray thought hydrastin and nux vomica would succeed well.

Dr. Holmes presented some interesting pathological specimens from the eye, one a melanotic, and another of which, he thinks, there are but six similar on record—a non-pulsating erectile tumor. This being removed, the eye resumed its original position in the orbit.

